# Medical Opinion and Movement

**Published:** 1907-02-09

**Authors:** 


### Medical Opinion and Movement.

The death of Sir Michael Foster took place suddenly on Tuesday night. Only the previous Saturday he was working in the laboratories at the Stansted farm of the Royal Commission on Tuberculosis, of which he was chairman, and on the previous Monday he was speaking at the annual meeting of the British Science Guild at the Mansion House. He studied medicine at University College, where afterwards he became teacher and then Professor of Physiology. Prior to this he was for a few years in general practice at Huntingdon. As Professor of Physiology at Cambridge he took a large share in the development of the Cambridge Biological School. His contributions to original research were not very great, but he exercised a profound influence as a teacher, both by his lectures and by his writings. He founded the *Journal of Physiology*, and his "Text-book of Physiology" is renowned as much for the charm and beauty of the style as for the exhaustive treatment of the subject. He was elected a Fellow of the Royal Society in 1872, and was created K.C.B. in 1899. In 1900 he represented the London University in the House of Commons as a Liberal Unionist, but later he became Liberal, and he lost his seat to Sir Philip Magnus at the last general election.

Urotropin has now enjoyed the favour of the profession for some years as an antiseptic drug for the urinary tract. It seems likely that it will now give place to the more complex chemical product called hetralin. Hetralin is a combination of resorcin and urotropin, and is represented by the chemical formula $C_6H_{12}N_4.C_6H_6O_2$. It depends for its antiseptic power, like urotropin, upon the liberation of formaldehyde in solution. This separation of formaldehyde takes place much more readily from hetralin than from urotropin, and can only be prevented by a reduction of the temperature to below 0° C. On the other hand, it is promoted by warmth and by acids. The resorcin component of the drug raises the acidity of the urine, and consequently assists the liberation of formaldehyde in alkaline or neutral urine. According to Wilhelm Fries, who has thoroughly investigated the subject, formaldehyde appears in the urine ten minutes after the administration of hetralin by the mouth, and can be detected for several days afterwards, varying according to the amount of the dose. The antiseptic power of hetralin has been shown to be considerable, at least *in vitro*. It is more powerful in acid than alkaline media, but even in the latter it is said to be much more efficacious than urotropin.

A case of considerable interest to the medical profession has recently been decided by Mr. Justice Warrington in a way which will doubtless be a matter of surprise to many. The parties to the suit, Clifford *v.* Timms, were dentists in partnership, but the questions involved might equally well arise between medical men. The plaintiff was summoned before the General Medical Council for employing unregistered assistants and publishing pamphlets of an objectionable character, and he was found "guilty of infamous or disgraceful conduct in a professional respect," and his name was directed to be erased from the Dentists' Register. In accordance with a clause in the deed of partnership, the defendant gave notice to determine the partnership. This clause provided that if either partner should be guilty of professional misconduct, or of any act which was calculated to bring discredit upon or injure the other partner or the partnership business, the other might by notice determine the partnership. The plaintiff brought an action on the ground that the notice was invalid. Mr. Justice Warrington found for the plaintiff. He held the opinion that the decision of the General Medical Council was not binding on him, and on the evidence before him he formed the conclusion that the plaintiff was not guilty of professional misconduct. There can be little doubt that the usual insertion of such a clause in partnership deeds is intended to cover any such decision of the General Medical Council. Nothing could more discredit or injure a partnership than the removal of the name of one of the partners from the register. In view, therefore, of the possibility of a court of law overriding a decision of the General Medical Council, all partnership deeds should contain a clause definitely making such removal a ground for the determination of a partnership.

In discussing measures for reducing the high mortality of infants, also for staying the ravages of tuberculosis, we have frequently laid stress on the importance of attempts to educate the people in the fundamental principles of the rearing and feeding of infants, and to give them some practical knowledge of the prevention and treatment of consumption and other infectious diseases. The Education Department of the London County Council is doing something on these lines by evening classes in "First Aid," "Home Nursing," "Infant Care," and "Health." We have on a previous occasion referred to the work of the Marylebone Health Society. This Society was formed a year ago to aid working mothers of the borough in the care and management of their infants, and to spread elementary knowledge of the prevention of consumption. The Society has arranged its third course of special lectures, which are to be given every Friday during this and the following month at 5 p.m., at 77 Welbeck Street. Subjects chosen include: "The Importance of Mastication," "The School Child," and "Some Points in Infectious Fevers," and the Society has secured for these lectures the services of such eminent men as Dr. Harry Campbell, Dr. B. L. Abrahams, and Dr. Sydney Phillips. The Honorary Secretary of the Lectures Committee is Miss Prince, of 22 Upper Wimpole Street, W. We feel sure that these educational endeavours are on the right lines. Hence we hope that they may meet with the success they deserve, and may stimulate like efforts in other districts.

